# 

**Published:** 1816-12

**Authors:** 


THE LONDON

### Medical and Physical Journal.

6 OF VOL. XXXVI.] DECEMBER, 1816. [NO. 214.

### ADDRESS.

*Some very good friends to our undertaking have addressed us lately on the frequency and length of communications on the same subject. If these gentlemen knew the numbers we decline to insert, compared with those which meet their eye, they would not fail to sympathize with us. Respecting the late controversy concerning Midwifery, few subjects required a more candid or minute inquiry; and, upon a re-perusal of the whole, we see no reason to regret the insertion of a single paper.*

*We hope our Nestorean Correspondent will not feel offended, if we offer a few words in favor of his communications. To us, his advice, though not all new, appeared to be all judicious, and, in many instances, likely to bring subjects before our younger readers, the importance of which they might not have appreciated. The practical remarks on Small-pox too, though not different from Sydenham's, are divested of his erroneous theory, and collect in a single point what is scattered throughout that great man's works. But our principal motive for gladly bringing the subject before our readers was, that the Small-pox now so rarely occurs, excepting in poor families, as to render many of the younger practitioners unsuspicious of its access, and sometimes at a loss in its treatment.*

*To conclude, the increasing encouragement we meet with, convinces us of the public satisfaction. In so much matter, collected twelve times in a year, the experienced practitioner will not expect to meet with novelty in every part. It must be recollected, that what is familiar to one set of readers may be new to another. In the figurative, but much stronger language than the ancient bard, who had only three guests to satisfy, we may plead,*

> Quid dem, quid non dem? Renuis quod tu jubit alter.
> Quod petis, id sane est, invisum acidumque duobus.

### CATALOGUE OF MEDICAL BOOKS.

Medico-Chirurgical Transactions, published by the Medical and and Chirurgical Society of London, Vol. VII. 8vo. 12s. boards.

Observations on the projected Bill for restricting the Practice of Surgery and Midwifery to Members of the Royal Colleges of London, Edinburgh, and Dublin, and to Army or Navy Surgeons. By a General Practitioner. 1s. 6d.

A Treatise on the Diseases and Organic Lesions of the Heart and great Vessels. By J. N. Corvisart, M.D. Translated from the French. 8vo. 10s. 6d.

Notices

### NOTICES TO CORRESPONDENTS.

Mr. Ring's *favour, though dated the 18th Nov., arrived too late for the present Number. If he will permit its curtailment, it shall, as he wishes, appear the first article in our next. Meanwhile we think it right to premise that there are passages in it which, we trust, will satisfy Dr. Kinglake; and that Mr. Ring informs us he has "cancelled that part of his work which was founded on erroneous information." Having said thus much, if Mr. Ring will not permit us to shorten his paper, we shall feel obliged to return it.*

*We have not been unmindful of the Communication from a* "Medical Officer;" *but many parts of his doctrine and practice were so new, that, coming, as the paper did, from an anonymous writer, we have not ventured to insert it without making very close inquiries of other "Medical Officers" in similar stations. Hitherto they have not confirmed our correspondent's practice.*

*We are ready to insert* Mr. C.'s *Lectures in the usual form. If he requires more, it must be in the form of an advertisement on our cover. However, the latter part of his communication may be admitted among our Original Communications, if drawn up in that form, with a more enlarged description of his improved instrument.*

*We are much obliged to* Mr. Spencer *for his Communication and Drawings, which will be inserted as soon as he will favour us with the title-page, and, if possible, the date of the book from which they are extracted: without these he will at once see that the paper is incomplete.*

*We feel every sentiment of gratitude to* Amicus *for his friendly hint. Our Correspondent at Paris, though much attached to physiology, is not, strictly speaking, professional; and his writing is often difficult. We shall, therefore, for the future, be more careful in correcting his papers after they come from the press.*

Alfred *is a very smart fellow, and gladly would we amuse our readers with his wit, as we have been ourselves amused; but it is a maxim with us not to admit anonymous remarks on papers to which the writer's name is affixed: besides this, Alfred will see that the writer whom he so much extols, and whose hard fate he pities so much, has an adversary who sticks pretty closely to him. We feel further obliged by Alfred's remarks, as they teach us to drop a controversy the interest of which must have ceased.*

*We have taken the liberty, for the above reason, to shorten the paper of our Correspondent from Rochdale; and shall also avail ourselves of his hint.*

*Communications have been received from* Mr. Harrison, Mr. W. S. Clarke, H. I. C., *&c. &c. most of which will appear in our next Number.*

### INDEX

### TO THE THIRTY-SIXTH VOLUME.

ABDOMINAL obstruction, two cases of 359
Abercrombie, Dr., his case of cynanche laryngea 52
Accoucheurs, on the necessity of 197
Accum, Mr., on the Moira saline water 428
Achyrantes, two species of that plant, with their virtues 344
Affinity, on vital 125
Africa, on the heat of 138
Agaricus campanulatus, deleterious effects of the 451
Air, effects of the growth of plants on the 80
Albers, Dr., his prize essay on Croup 306
Alcyonium, description of a fossil 212
Allan, Mr., on blood-letting in fever 246
Aloe perfoliata, medicinal virtues of the 342
Amenorrhœa, efficacy of ergot of rye in 380
America, prevalence of catarrhs in North 262
Amputation, nail re-produced after 19
——— at the shoulder joint, case of 108
———, new method of tying arteries in 227
——— at the instep, case of 355
ANALYSIS of new publications 46, 130, 218, 306, 392, 481
Ancients, on the surgery of the 448
Aneurism, method of tying the arteries in 227
——— from an old wound 331
Angina pectoris, two cases of 481
Angustura bark, poisonous effects of spurious 162
Animal functions, observations on the 122
——— heat, influence of the nervous system in 486
——— organism, effects of phosphorus on 338
Animals, on the dissection of living 196, 457
Annona, pungent and aromatic qualities of 344
Antimony, efficacy of the tartrite of 286
Aorta, production of a sac at the mouth of the 51
———, experiment on that of a dog 95
Aphony, effects of electricity in the cure of 164
Apothecaries company, their examinations in midwifery 284
Apothecaries' company, their regulations for practitioners 428
Arbutus uva ursi, medicinal virtues of 471
Aristolochy, odorous qualities of the 343
Armies, medical history of the British 233
Arsenic, on the tests for detecting 238, 239
Arteries, observations on the 94
———, on the powers of the 177
———, method of tying them in aneurism 227
———, on the ligature of the 235
———, two cases of wounded 332
Ashburner, Dr., on the saliva of rabid animals 337
Assalini, Professor, observations on his compressor 236
Asthma, effects of galvanism in 165
———, appearances, on dissection, in a case of humoral 331
Athlone, vote of thanks to Dr. Pitcairn, from the inhabitants of 335
Atkinson, Mr., his reply to Dr. Kinglake 96, 458
———, observations in answer to 288, 385
Authenrieth, Professor, on the causes of deafness 486
Bacopa, or burnweed, description of the 343
Baillie, Dr., letter to 20
Balfour, Dr. W., his method of curing rheumatism 318
———, his answer to the reviewer 445
———, reply to 447
———, on the re-union by the first intention 420
———, on the cure of gout ib.
Ball found in the heart of a buck 426
Ballota luna, its efficacy in dropsy 345
Barnes, Mr., his case of incontinence of urine 235
Barometer, the leech a natural 56
Barytes, on detecting arsenic by 239
Barton, Dr., on the pyrola umbellata and arbutus uva ursi 471
Bedingfield, Mr., his compendium of medical practice 402
Belladonna, its effects on the iris 267
———, its efficacy in hooping cough 435
Bethlem Hospital, elections at 81
Bibliography, medical 340
Bigelow, Dr., on the clavus or ergot of rye 376

### INDEX.

Bignana copaia, description of that tree and its virtues 342
Black, Do. Samuel, his cases of angina pectoris 481
Bladder, case of ulcerated 235
——, virtue of mountain sage in disorders of the 343
Blane, Sir Gilbert, on the health of the British navy 234
Bleeding, rabies cured by 71
—— efficacious in diabetes 115, 119
—— beneficial in high inflammation 179
—— —— palsy 194
——, preference given to topical, in several cases 364
—— in fever, advantages of 135, 153, 155, 246, 368, 371
—— useful in epilepsy 406
Blindness, observations on night 362
Blood, observations on arterial and venous 254
Boerhaavia, virtues of that plant 343
Bone, on the operation of nature in the formation of 157
Books, announcement of new medical 170, 176, 264, 437, 522, 524
——, catalogue of medical 88, 176, 264, 352, 439, 527
Borden, Dr., on the physiological theory of 340
Botanical excursions 84, 171, 258, 349
Botany, account of a pocket compendium of 170
Bottles, on dropping liquids from 426
Braid, Mr., his case of re-union of an amputated finger 420
Brain, dissection of the 437
Bread, on the adulteration of 429
Brenan, Dr., on abdominal obstruction 359
Brewster, Dr., on the eyes of fishes 166
Briggs, Mr., on the cutting gorget 407
——, Dr., his case of mortification of the intestines 249
Briony root, poisonous nature of the 435
Bristol, freedom of that city voted to Mr. Goldwyer 427
Brodie, Mr., on ulcerations of the joints 229
Bronchotomy, affections of the larynx that require the operation of 61
Brookes's Museum, remarkable skull in 254
Bulam fever, observations on the 142
Bull rush, uses of the 349
Burns, efficacy of cold water in 239
——, the bacopa beneficial in 343
Bursera gummifera, description of the ib.
Bush, Francis, on the hernia humeralis 286
Butter, methods of adulterating it, exposed 430
Cachexia syphiloidea, on the term of 333
Cadet, M., on the natives of Guiana 341
Cadiz, on the fever at 152
Cæsarean operation, success of the 198
Calculus, a biliary one of extraordinary size 69
——, a large one removed from the urethra 235
Cancer, on that of the womb 250
——, excision of the 293, 295
——, on different opinions concerning 459
——, cases of that of the breast 461
——, on the treatment of 464
Carpenter. W. H., his case of strangulated hernia 453
Cartilages of the joints, on the ulceration of the 229
Cascarilla, utility of the bark of 342
Cases of—puerperal fever, 2; midwifery, 6, 7; measles, 11; the re-production of a nail, 19; diabetes mellitus, 26; artificial pupil, 49; conversion of the substance of the heart, 51; cynanche laryngea, 52; affections requiring bronchotomy, 61, 63; obstruction in the intestines, 69; hydrophobia, 75, 79; intus susceptio, 86; hydrocephalus, 100; amputation at the shoulder joint, 108; diabetes treated by depletion, 115, 119; epidemic dysentery, 139; yellow fever, 146; aphony cured by electricity, 164; defective testicle, 174; inflamed testis, 178; the virtues of colchicum in chorea, 181; scrofula, 189; palsy, 194; rheumatism, 201, 203, 205; phthisis pulmonalis, 215; ulceration of the knee-joint, 231; hernia ventriculi, 232; calculus, 235; incontinence of urine, ib.; mortification of the uterus, ib.; mortification from swallowing a tooth, 249; intestinal disease, 250; pleuritis cured by poultices, 252; relief from injections into the uterus, 272; diseased vertebræ, 279; chorea, 292; cancer of the womb, 296; rheumatism, 323, 324; contraction of tha uterus, 331; humoral asthma, 332; sympathy between the eye and the intestines, 346; supposed poison, 350; amputation at the instep, 355; abdominal obstruction cured by spirits of turpentine, 359; palsy, 362; bleeding in fever, 369; croup, 373; amenorrhœa, 380; disorders of the eyes, 382; water in the head, 388; apparent death, 395; melancholy, 399; pharyngis ulceratio, 402; laryngis et tracheæ ulceratio, 405; inflammation of the large intestines, 418; a new practice in gout, 420; laryngitis, 421; poison from the briony root, 435; an affection of the eye from an uncommon cause, 442; hiccough cured by colchicum, 444; hernia cerebri, 449; the deleterious effects of agaricus campanulatus, 451; strangulated hernia, 453; cancer in the breast, 461; mortification in a whole family, 477; hydrophobia, 480; angina pectoris, 481; fatal affection of the pudendum, 482; uterine hæmorrhage, 497; deglutitio impedita, 512; secondary small-pox, 517; an iliac passion, 518; epilepsy cured by houseleek, 521.
Cassia, various species of 342
Cataract, success of galvanism in 510
Cayenne, account of plants peculiar to 342
Celsus on the cynanche 68
——, on a mistaken passage in 156
Champignons, fatal effects in a mistake of 451
Chaumeton, Dr., on legal and judicial chemistry 428
Chaussier, M., on deformity 219
Chemistry, on legal and judicial 428
Chichester store-ship, dreadful fever in the 146
Child-birth, comparative view of mortality in 199
Children, on the management of 220
——, fatal affection in female 482
Chopart's operation, remarks on 448
Chorea, efficacy of colchicum in 181
——, on purgatives in 425
——, observations on 511
Chrystallines, on venereal 482
Chyle, on the nature of 237
Chyme, observations on ib.
Clarke, Mr., his case of inflamed testis 178
Clavus, a disease peculiar to rye 376
Clutia rosæa, description of that plant 343
Coccolabo, two species of that plant described 342
Colchicum not a new remedy in gout 95
——, its efficacy in chorea 181, 292
——, obstinate cough cured by 444
Coleridge, Mr., has the small-pox twice 517
Collectanea Medica 33, 114, 207, 293, 376, 466
Collins, Dr., on the virtues of marine plants 182
Collingwood, Dr., on diseased vertebræ 279
Consumptions, observations on the frequency of 214
Contagion, remarks on 138, 372
Controversy, inconvenience of medical 365
Convolvulus macrochizos, description of the 343
Coombe, T. G., his case of hernia cerebri 449
Copaifera officinalis, the balsam produced by 343
Copper vessels dangerous in cookery 431
Correspondents, notices to 88, 176, 264, 352, 438, 528
Cough, obstinate one cured by soda 373
——. cured by colchicum 444
Contaucean, M., on physiology 81
——, chemical observations of 341
Crawford, M., his cases of wounded artery 332
—— on humoral asthma ib.
Cross, Dr., his case of asthma 194
Croup, on the causes of 306
——, efficacy of potass in 373
Cullen, Dr. on bleeding in fever 135
——, observations on his theory of gout 500
Cupping, a new machine for 83
Cynanche, observations on the acute 67
—— laryngea, case of 52
Cynanchum, a plant of Guiana, described 343
Davies, Rev. H., his observations on the sea long-worm 207
Deafness, prize question on 254
——, on the causes of 436
Deformity, observations on 219
Deglutition, on impeded 512
Denmark, Dr., on the Mediterranean fever 155
Dentition, observations on 222
Depletion, its advantages in fever 368
Diabetes mellitus, case of 26
——, benefit of opium in 30
——, on the urine in 31
——, efficacy of bleeding in 115
Diagnosis, contributions to 417
Diaphragm, fatal case of lacerated 233
Dickson, Dr., his reply to the reviewer 130
——, answer on the depletory treatment of fever 368
Digestion, on that of cold animals 58
Digitalis, its effect in puerperal fever 1
——- used in hydrocephalus 101

Diseases, monthly reports of 86, 174, 261, 350, 439, 526
———, on contagious 138
———, on vegetable 377
Dissection, on that of living animals 196, 457
——— of the brain 427
Distemper, on the term 73
Dogs, advice on the management of 71
——, experiment on the aorta of one 95
——, on the wounds of 169
Doses, tabular view of the proportions of 104
Doughty, Mr., on the yellow fever 140
Drops in medical practice, rules concerning 426, 516
Dropsy, remedy used in Siberia for the 346
———, on the blood of patients in 515
Dublin, deaths from child-birth in the Lying-in Hospital at 199
———, cases of hydrophobia at 480
Dunstan, Mr., his account of St. Luke's Hospital 35, 40
Duval, M., on the preservation of the teeth 255
Dysentery, topical bleeding recommended in 86
Dzondi, Dr., on cold applications in burns 239
Ear, cause of disorders in the 436
Earle, Mr. Henry, on animal heat 486
Eau medicinale, efficacy of the 506
Editor, address of the 441
Edinburgh, prize question of the Medical Society of 80, 254
——— Medical Journal, review of the 47, 246, 410
———, dissection of the brain at 427
Education, observations on physical 218
Electricity, case of aphony cured by 164
———, its effects on meat 170
Emmert, Professor, on the Angustura bark 162
English, Mr., on warm poultices in inflammation 251
Epidemics at the Cape of Good Hope 178
Epilepsy, observations on 406, 472
———, houseleek efficacious in 521
Ergot, extraordinary properties of that of rye 376, 474
Eruptions after vaccination 17
Eryngium fetidum, its medical qualities 344
Eye, artificial pupil in the 49
—, causes of inflamed pupil of the 89, 265
—, telescopic power of the 91
—, on that of fishes 166
—, on rheumatism in the 200, 382
—, its sympathy with the intestines 346
Eye, on various disorders incidental to the 382, 386
—, effects produced by infection from sore 415
—, singular affection of the 412
Eye-stones, superstition in America concerning 82
Fat, observations on the formation of 303
Females of southern Africa, observations on a singular affection in young 482
Fever, the puerperal, treated by injections 1, 272
——, advantages of bleeding in 130, 135, 246, 369
——, on that of Bulam 140
——, observations on that of the Mediterranean 155
——, on the secondary recurrence of the yellow 330
——, observations on puerperal 512
Finger, nail produced after amputation of the 19
——, re-union of a 169, 420
Fishes, on the eyes of 166
Flap operation, case of 355
————, observation on the 448
Flies, observations on the feet of 253
Flora, the calendar of 84, 170, 248
Fluids, on dropping them from bottles 426, 516
Fluor albus, description of that disorder 543
Foramen sacra sciaticum, hernia in 518
Fothergill, Dr. Samuel, his arrival in Jamaica 437
French Pharmacopœia, account of a new 522
Friedlander, Dr., on physical education 218
Frogs, on the formation of fat in 165
——, account of one killed by leeches 57
Fucus marinus, description of the 184
—— vesiculosus, preparations of 186
—— nodosus described 190
Fumigation, on that of letters 253
Functions, on the animal 122
Galibis, practice of physic among the 341
Galvanism, its efficacy in nervous diseases 165
Garangeot on the restoration of lost noses 169
Gaubius, on the table of 105
Glasgow, medical degrees taken there 257
Glen, Mr., on the deleterious effects of agaricus campanulatus 451
Goden, Dr., on hydrophobia 75
Goldwyer, Mr., freedom of Bristol voted to 427

Gomez, Dr., on fumigating letters 253
Gordius marinus, description of the 207
Gordon, Dr., on the buffy coat of the blood 254
Gorget, memoir on the cutting 407
Gout, colchicum not a new remedy in 95
——, new method of treating the 420
——, on the nature of 498
Graham, Mr., his case of mortified uterus 235
Grain, on the preservation of 377
Grainger, Mr., on a ball found in the heart of a buck 426
Gratiola, the properties of 342
Green, Dr., on the virtues of turpentine in abdomical obstruction 359
Gregory, Dr., on measles 12
Guiana, medical practices in 341
——, medicinal plants in 342
Gumprecht, Dr., on lactuca virosa in hooping cough 235
Hæmorrhage, on uterine 489, 514
Halford, Sir Henry, on insanity 33
Hall, Dr. John, on bleeding in fever 368
—— Dr. Marshal, his contributions to diagnosis 417
Hands, on the swellings in the tops of the 425
Harveian oration, account of the 516
Hawkins, on the cutting gorget of 407
Haworth, Dr., his Harveian oration 516
Hayti, letter from the king of 167
Head, case of water in the 388
Heart, case of conversion of the substance of the 51
——, on a disease of the valves of the 52
——, on the voluntary command of the 93
——, ball found in that of a buck 426
Heat, influence of the nervous system on animal 486
Heberden, Dr., his case of watery head 388
Heischman, Dr., account of his dissections 515
Hellebore, its virtues in gout 506
Hemlock, its effects in cancer 462
Hernandia, a purgative plant of Guiana 342
Hernia ventriculi, case of 232
—— cerebri, case of 449
—— humeralis, antimony a remedy for 286
——, case of strangulated 453
Hey, Mr., his claim to the flap operation 448
——, his observations on midwifery 99
Hiccough, case of obstinate, cured by colchicum 444
Hill, G. N., his case of disease in the intestinal canal 250
—— Dr., his case of laryngitis 421
Hoare, Mr., on vaccination 9
Home, Sir Everard, on the feet of flies 253
——, on the fat of frogs, &c. 165
——, observations on his theory 303
Home, Dr., his practice of inoculating for the measles 13
Hooping-cough, efficacy of soda in 373
——, belladonna said to be useful in 435
Hop, on the medical properties of the 456
Hottentots, description of the female 466
Houseleek, its virtue in epilepsy 521
Howship, Mr., on the formation of bone 157
Hudibras, Latin translation of 197
Hume, Mr., on the detection of arsenic 239
Hunter, John, his error concerning the leech 60
——, on adhesive inflammation 315, 477
——, ignorance of the practitioners on the continent respecting his doctrines 517
Hura crepitans, an American plant, described 342
Hydrocephalus, successful case of 100
——, on the use of digitalis in 103
——, method of treating 388
——, operation performed in sheep for 428
Hydrophobia, cases of 71
——, new method of treating 74
——, caustic ineffectual in 435
——, cases at Dublin of 479
——, dissections in cases of 517
——, benefit of the meloe proscarabæus in 519
Hypericum sessilifolium, description of the 343
Hypochondriasis, essays on 392
Hyssop, virtues of the hedge 506
Icica, an American balsamic tree 345
Indians, practices of American 98
Infants, on giving opiates to 417
Infection, means of preventing 253
Inflammation, on bleeding in high 178
——, warm poultices recommended in internal 251
——, on adhesive 315
——, insidious progress of internal 335
——, prevalence of 350
——, effects of turpentine in preventing 359

Inflammation of the intestines, case of 418
Inflammatory affection of the throat 47
Influenza, disorder resembling the 262
Injections recommended in puerperal fever 1
———— forms of, for the uterus 272
Inoculation for the measles, account of 13
Insanity, evidence before the House of Commons on 33
————, observations on 392
Insects, tabular view of 209
————, on the feet of 253
————, grain affected by 377
Instep, case of amputation at the 355
INTELLIGENCE, Medical and Philosophical 71, 164, 253, 335, 426, 516
Intestinal disease, case of remarkable 250
Intestines, obstruction in the large 69
————, sympathy of the eye and the 346
Iris, observations on the 90
Irritability, observations on 124
Jackson, Dr. James, on the properties of life 120
————————, on rheumatism of the heart 200
Jacksonian price for 1817, subject of the 254
Johnstone's, Dr., natural history of the medicinal leech 52
Joints, on ulcerations of the cartilages of the 229
Jones, Dr. E. G., on the mineral waters of Spa 481
Kelly, George, on swellings of the hands and feet 425
Kennels, directions for the management of 71
Kerr, Mr., on the power of arteries 94
————, on colchicum in gout 95
————, answer to him on the powers of arteries 177
King, Mr., on inoculating rabid matter 337
Kinglake, Dr., on obstetric practice 3
————————, reply to him on the same 96
————————, his answer 288
————————, copy of his diploma 253
————————, his inaugural dissertation 355
————————, observations on his communications 365
————————, answer to 458
Knight, Mr., his experiments on the leaves of plants 166
Knowles, E. L., his case of violent contraction of the uterus 331
Language, on nosological 329
Laryngis ulceratio, case of 404
Laryngitis, cases of 421
Larynx, on some affections of the 61
Latham's, Dr., case of bronchotomy 65
Lawrence, Mr., on bronchotomy 61
————————, on tying the arteries 227
————————, observations on his lectures 425
Leach, Dr. his tabular view of insects 209
Leaves of plants, experiments on 166
LECTURES announced—Dr. Clough on midwifery, 84; Mr. Salisbury on botany, 85; Drs. Latham and Southey on the practice of physic, 173; Drs. Squire and Davis on midwifery, ib.; at St. Bartholomew's Hospital, 260; at St. Thomas's and Guy's Hospitals, ib.; at St. George's Hospital, ib.; at the University of Glasgow, 261; Dr. Adams on the institution and practice of medicine, ib.; Dr. Clutterbuck on the theory and practice of physic, &c. ib.; Dr. Collingwood on the theory and practice of medicine, ib.; Mr. Wilson and Mr. Bell on anatomy and surgery, ib.; Mr. Clarke on midwifery and the diseases of women and children, ib.; Dr. G. Currey on the practice and theory of medicine, ib.; Mr. Taunton on anatomy, &c. 347; Dr. Clough on midwifery, ib.; Dr. Davis on the same, ib.; Dr. Merriman and Dr. Hey on the same, 347, 524; Mr. Guthrie on medical and operative surgery, 348.
Lecythis omara, a plant of Guiana, described 344
Leeches, natural history of 52
————, substitute proposed for 83
————, caution in the use of 463
Letters, method of fumigating 253
Lewes, fossil alcyonite found at 212
Lichtenstein's Travels in Africa, extract from 138
Life, on the science of 81
—, on the properties of 120
Ligature of the arteries, on the 235
Lightning, its effects on sheep 169
Lime, on the crystallization of 255
Liniment made from sea-weed 188
Linnean Society, anniversary meeting of the 167
Liquids, regulation respecting the measure of 426, 516
Liquors, methods of adulterating 431
Lithgow, Dr., his case of chorea 425
Littleton, Mr., on the causes which influence the pupil of the eye 89, 265
Lizards, observations on 58

Lobstein, M., on the virtue of phosphorus 337
London, mortality from child-birth in 199
Luke's, St., Hospital, particulars concerning 35
Lunacy, evidence before the House of Commons on 33
Lupulus, on the medicinal uses of 456
Lysianthus, an American plant, described 344
Macgrigor, Sir James, on phthisis pulmonalis 214
——————, his medical history of the British army 233
——————, observation on 249
Mad-houses, evidence before the House of Commons on 33
Makesy, Mr., his observation on ophthalmia 415
Malpighia, virtues of the bark of 344
Mangioli, M., on the venom of the viper 522
Mantell, Mr., his account of a fossil alcyonite 212
Marantu arundinacea, or arrow plant, described 342
Marcet, Dr., on the nature of chyle 237
————, on tests for arsenic 238
Marine plants, observations on 182
Mauriceau on puerperal fever 2
Mayow, Dr., his discovery of the muscles 251
Measles, observations on 10
————, on the inoculation of 13
Medicago arborea, an American tree, described 343
Medical and Philosophical Intelligence 71, 164, 253, 335, 426, 516
——— Society of Edinburgh, question of the 80, 254
——— students, advice to 192, 508
——— bibliography, account of 340
Medicine, table of the doses of 104
——— review of the state of 410
Medico-chirurgical remarks and observations 510
——————— Transactions, analysis of the 61, 155, 227, 481
Mediterranean, observations on the fever of the 155
Melancholy, cases of 399
Meloe proscarabæus, or May-bug, beneficial in hydrophobia 519
Merriman, Dr., answer to 3
Meteorological register 87, 175, 263, 351, 438, 525
Midwifery, controversy concerning the practice of 3, 96, 197, 288, 365, 458
————, cases in 272
————, on the proposed regulation of 283
Milk, on the adulteration of 430
Miller, Mr., his case of palsy 362
Minim, on the measure of the 426, 516
Mirabilis, virtues of the root of the 343
Moira saline water, analysis of the 428
Momordica balsamica, an excellent vulnerary 343
Monro, Dr., reply to him on the discovery of oblique muscles 251
Moore, Mr., on an artificial pupil 49
———— letter from the King of Hayti to 169
Morgagni on a case of hydrocephalus 391
Mortality from child-birth 199
Mortification of the intestines, case of 249
————— uterus 235
Murray, Mr., his account of an epidemic sore throat 47
Muscles, on the discovery of the oblique 251
Mushrooms, danger of mistakes in 451
Myradendron balsamifera, on the resin of 343
Nail, case of one re-produced after amputation 19
Navy, comparative statement of the health of the 234
Nervous diseases, efficacy of galvanism in 165
——— affections, essays on 397
——— system, its influence in regulating animal heat 486
Nitrate of silver a test for arsenic 238
Nixon, Dr., on a case of aphony cured by electricity 164
Nose, on the restoration of a lost 169
Nosology, outlines of a new system of 327
Novels, utility of 398
Nuckel, Dr., on hydrophobia 518
Nurses for the sick, regulations in France of 520
Nyctalopia, or night blindness, observations on 362
Oblique muscles, on the discovery of the 251
Obstetric practice, controversy on the 3, 96, 197, 288, 365
——————, effects of the Surgeons' bill on 283
Obstruction in the large intestines 69
————, cases of abdominal 359
Ocymum, varieties of that plant 345
Omphalia diandra, its efficacy in ulcers 345
Ophthalmia, infectious nature of 413
Opiates dangerous for children 417
Opium, its efficacy in contracted uterus 331
———— dangerous in nervous diseases 398
Oracular communications 508

### INDEX.

Orelia allamanda, a medicinal plant, described 343
Organs, necessity of studying the 81
Organism, effects of phosphorus on animal 338
Origanum majorana, pungency of the 345
Osiander, Professor, his mode of curing cancer 250, 293, 295
Oxalic acid, persons poisoned by 517
Painciana, or peacock flower, an American plant 343
Palsy, remarkable cases of 194, 362
——, its effects on the eye 269
Paralysis, causes of 270
Parkinson, Dr., his system of nosology 327
Parrot, on the iris of the 93
Parry, Dr., on the powers of the arteries 177
Paullina pinnata, an excellent vulnerary 315
Pearson, Dr., on diseases of the heart 52
Percy, M., his experiments on dogs 169
Peters, Dr., on the virtue of houseleek 521
Pharmacopœia, account of the French 522
Philip, Dr. W., answer to 46
—————— on nervous diseases 165
Phosphorus, on the healing virtues of 337
——————, its effects on the animal system 338
Phthisis pulmonalis, observations on, in warm climates 214
Physiology, advantages of the study of 81
Phyllanthus, an American plant, described 344
Phylotacca, medicinal virtues of the 343
Physicians, regulation of the College of, in dispensing medicines 426
——————, anniversary of the 517
Piper trifolium, its virtues 343
Pipes, on the composition of water 430
Pitcairn, Dr., address of thanks to 335
Placenta, on the best mode of extracting the 286, 363
Plants, effects of their growth on the atmosphere 80, 252
———, experiments on the leaves of 166
——— growing round London 84, 171, 258, 348
———, on the virtues of marine 182
———, on the ergot in 376
Portuguese, superstition of the 198
Poultice, its efficacy in internal inflammation 251
Pudendum of female children, extraordinary affection of 482
Puerperal fever, injections recommended in 1
——————, observations on 512
Pupil, case of artificial 49
——, on the causes which influence the size of the 89, 265
Purton, Mr., on extracting the placenta 363
Pyrola umbellata, on the medical properties of the 471
Questions, prize 80, 254, 432
Rabies, methods of preventing 71
——, cases of 74
——, remarks on 479
——, the actual cautery the only cure for 435
——, dissections in cases of 517
Raven, Mr., on the virtues of colchicum in chorea 181, 292
——————, his case of obstinate hiccough 444
Reid, Dr., on hypochondriasis and nervous affections 392
Report of the National Vaccine Establishment 167
Re-union of a wound, observations on 445
Reviewer in the Medical Journal, his answer to Dr. Philip 46
——————, reply of Dr. Dickson to 130
——————, his answer to the same 135
——————, reply of Dr. Balfour to 445
Rheumatism of the heart, observations on 200
——————, new method of relieving 318
—————— of the eyes, cases of 382
Ring, Mr., correction of a mistake by 353
Riverius on injections in puerperal fever 2
Roberton, Dr., his letter to Dr. Baillie 20
Ronalds, Dr., his observations on measles 10
Royal Society, proceedings of the 164, 253
Rubeola, three species of 12
Rumford, Count, his bequest to Harvard College 83
Rye, on the ergot in 376, 474
Salisbury's, Mr., botanical excursions 84, 176, 258, 348
——————, account of his Botanical Compendium 170
——————, on useful plants 349
Satterley's, Dr., cases of diabetes 115
Scarlatina, prevalence of 86, 174
Scarpa, Antonio, on the cutting gorget 407
Scirrhus, observations on 459
Scudamore, Dr., on the nature and cure of gout 493

Serres, Dr., on a case of aphony 164
Shath, Dr., on a method of treating puerperal fever 1
——, his cases in midwifery 272
Sheep killed by lightning 169
——, operation on them for hydrocephalus 428
Sheppard, J. B., on yellow fever 330
Shoulder-joint, case of amputation at the 108
Siberia, method of curing the dropsy in 346
Sight, on weakness of 384
Skull, remarkable disease in a 254
Sleep, remedy for want of 398
Small-pox, axioms on the 14
——, remarks on Mr. Walker's paper on the 112
——, reply of Mr. Walker on 284, 458
——, answer to that gentleman 375
——, its ravages in London 350
——, secondary case of 517
——, recent cases of 526
Smerdon, C. W., his case of diabetes mellitus 26
——, on the distinction between diabetic and healthy urine 31
Somerville, Dr., his observations on the female Hottentots 466
——, on the virtues of the pyrola umbellata 471
Spa, analysis of the mineral waters of 481
Spallanzani, his remarks on the lizard 58
Spen, Dr., his case of conversion of the substance of the heart 51
Spiders, observations on 211
Sprains, new method of curing 318
Spurzheim, Dr., his dissection of the brain 427
Squire, Dr. John, death and character of 257, 347
Stammering, observations on 226
Stevenson, Mr., on the sympathy between the eye and the intestines 442
Stewart, Duncan, on uterine hæmorrhage 489
Stone, history of the first operation for the 519
Stones which have a remarkable effect on the eyes 82
Students, advice to medical 192, 508
Study, imprudence of excessive 397
Suck, Dr., his case of retroversion of the pupil of the eye 346
Sulphur, virtues of the liver of 373
Surgeons, prize questions of the College of 254
——, observations on their bill 283
Surgery, on that of the ancients 448
Surgical operations, account of 227
Sydenham, Dr., on measles 11
Sympathy, on the property of 129
—— of the eye and intestines 346, 442
Table of doses of medicine 104
—— of insects 209
—— of consumptive cases 217
—— for measuring fluids 426
Tadpoles, on the formation of fat in 165
Tartrite of antimony, its efficacy in hernia humeralis 286
Taunton, Mr., inscription on the cup presented to 337
Teeth, on the preservation of the 255
Telescopic powers of the eye, causes of the 91
Testis, case of reduced 174
—— inflamed 178
Thilenius, account of his Medico-Chirurgical works 510
Thomas, H. L., his case of obstruction of the intestines 69
Throat, case of inflammatory affection of the 47
Tooth, case of mortification from swallowing a 249
Travers, Benjamin, on the ligature of arteries 235
Trees, experiments on the sap in 166
Turpentine, spirits of, effectual in abdominal obstruction 359
Ulceration of the joints, on the 229
Ulcers of the groin, remedy for 189
Urethra, case of calculus removed from the 235
Urine, distinction between diabetic and healthy 31
——, case of incontinence of 235
Uterine hæmorrhage, observations on 489, 514
Uterus, cases of relief from injections into the 1, 272
——, on the excision of the 295
——, case of violent contraction of the 331
Uwins, Dr., his case of hydrocephalus 100
Uva ursi, observations on 472
Vaccination, statement of the practice of 9
——, eruptions after 17
——, report on 167
——, its success in St. Domingo 169
——, small-pox after 174
Vegetables, on the diseases of 377
Venereal disease cured without mercury 517
Venæsection, cases of wounded artery in 331

### INDEX.

Venæsection, preference of local bleeding to 364
Vertebræ, cases of diseased 279
Vessels, on the action of 339
Viper, experiments on the venom of the 522
Vivification, on the term 127
Volition, its influence on the iris 93
Wadd, Mr., on the formation of fat 303
Walker, Dr. John, on the surgeons' bill 283
——, Richard, on the small pox 14
————, his table of doses of medicine 104
————, answer to him on small-pox 112
————, his reply 285
————, rejoinder to 375
————, his answer 458
————, on scirrhus and cancer 459
————, on medical measures 516
Ward, Henry, on the dropping of fluids 426
————, on the medical properties of lupulus 456
Warren, Dr., on diseases of the eyes 382
Water in the head, case of 388
—— pipes, on the construction of 430
Wayte, Mr., answer to him 3
Wheat, on the ergot of 381
Wheelwright, Mr., his case of hernia 232
Whitridge, Dr., his case of amputation at the shoulder 108
Willan, Dr., on rubeola 12
Wines, on the adulteration of 431
Womb, on the cancer in the 250, 293
Wood, Mr., on an affection of the pudendum 482
Woodham, Mr., on the small-pox 112
————, reply to 284
————, his rejoinder 375
————, review of his remarks on Mr. Lawrence's lectures 425
Worm, description of the sea long 207
Wound, on the re-union of a 445
Yeates, Dr., on the discoveries of Mayow 251
Yeatman, Mr., his case of amputation at the instep 355
Yelloly, Dr., his case of calculus 235
Yellow-fever, observations on 140, 330
Young, Mr., on his method of healing cancer 460, 464

---

### PLATE.

Mr. Littleton on the Pupil of the Eye, page 265.

